# RETRACTION: Effect of Stem Cell Treatment on Burn Wounds: A Systemic Review and a Meta‐Analysis

**DOI:** 10.1111/iwj.70423

**Published:** 2025-03-26

## Abstract

**RETRACTION:** QiaoY. , ZhangQ. , PengY. , QiaoX. , YanJ. , WangB. , ZhuZ. , LiZ. , and ZhangY. , “Effect of Stem Cell Treatment on Burn Wounds: A Systemic Review and a Meta‐Analysis,” International Wound Journal 20, no. 1 (2023): 8–17, 10.1111/iwj.13831.35560869 PMC9797938

The above article, published online on 13 May 2022, in Wiley Online Library (http://onlinelibrary.wiley.com/), has been retracted by agreement between the journal Editor in Chief, Professor Keith Harding; and John Wiley & Sons Ltd. Following an investigation by the publisher, all parties have concluded that this article was accepted solely on the basis of a compromised peer review process. The editors have therefore decided to retract the article. The authors did not respond to our notice regarding the retraction.

**RETRACTION:** Y. Qiao , Q. Zhang , Y. Peng , X. Qiao , J. Yan , B. Wang , Z. Zhu , Z. Li , and Y. Zhang , “Effect of Stem Cell Treatment on Burn Wounds: A Systemic Review and a Meta‐Analysis,” International Wound Journal 20, no. 1 (2023): 8–17, 10.1111/iwj.13831.35560869 PMC9797938

The above article, published online on 13 May 2022, in Wiley Online Library (http://onlinelibrary.wiley.com/), has been retracted by agreement between the journal Editor in Chief, Professor Keith Harding; and John Wiley & Sons Ltd. Following an investigation by the publisher, all parties have concluded that this article was accepted solely on the basis of a compromised peer review process. The editors have therefore decided to retract the article. The authors did not respond to our notice regarding the retraction.

